# Corns Cured

**Published:** 1876-02

**Authors:** 


*Corns Cured.*

We stated in another number, that the simplest, safest, most available, and consequently the best cure for corns on the toes, consisted in three operations,—First: Soak the feet in hot water for fifteen minutes, night and morning, for a week.

Second: After the soaking, rub a little sweet oil on the corn, or any other mild form of grease, with the finger, for about five minutes.

Third: Cut a hole in one, two, or three thicknesses of soft buckskin, and bind it on the toe, so that the hole in the buckskin shall receive the corn.

The object of the water and oil is to soften the corn and parts adjoining; the object of the buckskin is to protect the corn from pressure.

In a very short time the corn will become painless, and will subsequently fall out of itself, as it is a growth, and is pushed upwards and outwards by the more natural growth beneath. It is thrown out of the body by the action of the parts, as a splinter or a crushed bone, as being no longer a part of the body. If anything else is done to the corn, it should be simply picked out with the finger nail, as cutting it makes it take deeper root, and dangerous bleedings sometimes occur when the knife is used. Our object was to recommend something which we knew to be safe, practicable, and efficient. We stated further, that corns, like consumption, were never cured; and that the original causes of them, to wit, pressure with a tight shoe, or friction with a loose one, would cause them to return, and even more readily than at first, because the structure of the skin about a corn is malformed for life, and would no more become entirely natural than a finger would grow again, if cut off. So, when a person has had consumption and gets well, he cannot be said to be perfectly cured, because he will always remain with a deficiency of lungs; the portion destroyed never can be replaced, yet, by making the remainder of the lungs work more fully, he may have even better health than he ever had before. Thus, it is literally and strictly true, that corns and consumption are never cured—because their very existence depends on the destruction of the organization and substance of a part of the body, never to be replaced.

There are corn doctors and corn salves; some of which latter are of very powerful constituents. They do remove the corns, but for no longer time than the plan we have advised, while ours has the preference, as costing nothing, and being wholly without danger. Remedies are valuable for the multitude in proportion as they are safe, cheap, and attainable.

If corns are pared down, from time to time, they widen their base, become more deeply rooted, and in old age blacken, cause gangrene and ultimate death! Therefore, we advise the prompt treatment of corns as above. It may be many months before they return; but when they do, the same treatment will always be effectual, and always safe.

